# Morbimortalidad de las infecciones del tracto respiratorio inferior y vacunación

**DOI:** 10.23938/ASSN.1063

**Published:** 2023-12-26

**Authors:** Manuel García Cenoz, Iván Martínez Baz

**Affiliations:** 1 Gobierno de Navarra Departamento de Salud. Instituto de Salud Pública y Laboral de Navarra Pamplona Spain; 2 Instituto de Investigación Sanitaria de Navarra (IdiSNA) Pamplona Spain; 3 CIBER Epidemiología y Salud Pública (CIBERESP) Spain

Sr. Editor:

Las infecciones del tracto respiratorio inferior (ITRI), que incluyen las neumonías y la bronquiolitis, son una causa importante de morbilidad y mortalidad.

En 2021 se publicó en Anales del Sistema Sanitario de Navarra el estudio realizado por Leache y col analizando la incidencia de hospitalizaciones debidas a ITRI en España, entre 1997 y 2018, a partir del Conjunto Mínimo Básico de Datos-Hospitalización (CMBD-H) y del Registro de Atención Especializada (RAE-CMBD)^1^. Este estudio nos muestra como las ITRI causan un 3,5% del total de ingresos hospitalarios anuales, siendo la neumonía la causa más frecuente de hospitalización por infecciones de las vías respiratorias bajas, acumulando hasta el 71% de los ingresos, principalmente en las personas de 65 años o más. Además, de todos los ingresos por neumonía, el 40% correspondieron a personas mayores de 75 años, así como el 76% de las muertes por esta causa.

Aunque el CMBD no recoge el agente causante de las diferentes ITRI, es conocido que diferentes microorganismos pueden causar neumonías, siendo las más frecuentes las producidas por *Streptococcus pneumoniae* (neumococo) y *Haemophilous influenzae*^2^. Entre las neumonías virales, el virus de la gripe, el rinovirus, el virus respiratorio sincitial (VRS) y el metapneumovirus son los agentes causales más frecuentes^3^. En 2020 se descubrió el nuevo coronavirus SARS-CoV-2, causante de neumonías de gravedad durante la pandemia de COVID-19, y que originó en su primera onda pandémica una importante morbimortalidad, con una tasa de defunción por COVID-19 confirmado de 0,8 por 1.000 habitantes, que superó el 1 por 1.000 en el grupo de las personas mayores de 75 años, y alcanzó el 1% en mujeres y el 1,4% en hombres mayores de 85 años^4^.

La bronquiolitis es un cuadro clínico que puede ser grave en los niños pequeños, especialmente en los menores de un año de edad, y en las personas de mayor edad. En el estudio de Leache y col^1^, el 40,7% de los ingresos por bronquiolitis se produjeron en menores de un año, seguido de los mayores de 74 años (27,1%). En el 60-70% de los casos es una complicación de la infección causada por el VRS, seguido por otros virus estacionales como el rinovirus, bocavirus humano, adenovirus y metapneumovirus^5^. La infección por VRS constituye la causa más frecuente de hospitalización en menores de un año en los países desarrollados. En la temporada de circulación del virus, hasta el 10% de los menores de un año pueden padecer una bronquiolitis, con la sobrecarga asistencia que conlleva tanto en Atención Primaria como en Urgencias. De ellas, hasta el 2% pueden requerir ingreso hospitalario^6^.

El estudio de Leache y col^1^ concluye citando la necesidad de intensificar medidas e intervenciones específicas para reducir la carga de ITRI, especialmente para las personas con mayor riesgo de sufrir complicaciones^1^. En la actualidad existen varias herramientas preventivas para minimizar los casos graves por estas causas. Por un lado, la vacunación con vacuna neumocócica conjugada, la cual se administra -de acuerdo al calendario de vacunaciones de Navarra- a todos los niños a los 2, 4 y 11 meses de edad, así como a los adultos que presentan ciertas condiciones de riesgo y a las personas institucionalizadas. Además, a los 65 años de edad se administra la vacuna polisacárida de 23 serotipos. Desde este año, se dispone de nuevas vacunas neumocócicas conjugadas de 15 y 20 serotipos (Vaxneuvance® y Apexxnar®, respectivamente), que podrán contribuir a reducir en mayor medida la carga de enfermedad por los serotipos cubiertos por la vacuna.

La vacunación anual frente a la gripe a las personas de 60 años y más, así como a otros grupos de población que presentan un mayor riesgo de complicaciones por esta infección, y -desde esta temporada 2023-2024- a los niños entre 6 y 59 meses de edad, es la mejor medida preventiva disponible para disminuir la incidencia de la enfermedad y, especialmente, para evitar formas graves de la misma. Se estima que en la temporada 2019-2020, en la que se alcanzaron coberturas de vacunación antigripal superiores al 60% en los mayores de 65 años, se pudieron evitar aproximadamente 990 consultas en atención primaria, 207 hospitalizaciones, 28 ingresos en UCI y 175 defunciones debidas a la gripe. Se estima que por cada 121 dosis de vacuna antigripal administradas se previno una consulta de atención primaria, por cada 580 dosis un ingreso hospitalario, y por cada 685 dosis una muerte^4^.

La vacunación frente a la COVID-19 supuso un antes y un después en la pandemia. Se alcanzaron coberturas de primo vacunación superiores al 90% y se redujo considerablemente la proporción de casos que requerían hospitalización o que fallecían^7^. En la temporada 2023-2024, se seguirá vacunando a la población que presenta un mayor riesgo de complicaciones derivadas de la infección por SARS-CoV-2, administrando una dosis de vacuna adaptada a la variante circulante en la actualidad (Omicrón XBB.1.5.)^8^.

Desde esta temporada, 2023-2024, se dispone de una nueva herramienta preventiva, un anticuerpo monoclonal, nirsevimab (Beyfortus®) destinado a la inmunización de los recién nacidos, así como a los menores de un año con alto riesgo de padecer la infección, como son los prematuros, así como otros lactantes que padecen condiciones que aumentan el riesgo de padecer enfermedad grave por VRS^9^.

Nirsevimab se administra en una única dosis intramuscular de 50 mg para los lactantes de menos de 5 kg, o de 100 mg para lo que pesen 5 kg o más. Los ensayos clínicos mostraron una eficacia del 85% para prevenir la infección por VRS, y del 89,4% para prevenir las hospitalizaciones. La reacción adversa más frecuente es la erupción (0,7%) que, según datos de la ficha técnica, se produce en los 14 días posteriores a la administración de la dosis, y suele ser de carácter leve o moderado. Además, se puede producir fiebre y reacciones en el lugar de la inyección en un 0,5% y 0,3% de los casos, respectivamente, dentro de los siete días posteriores a la administración de la dosis^10^.

En conclusión, una parte importante de la morbimortalidad debida a enfermedades del ITRI, y especialmente la gripe, puede ser evitada mediante la inmunización/vacunación de las personas con mayor riesgo de enfermar, de acuerdo a las recomendaciones de los programas de vacunación.
